# The Vaccination Papers a Waste of Time and Space

**Published:** 1898-08

**Authors:** S. Mills Fowler

**Affiliations:** Miami, Florida


EDITOR OF THE HOMŒOPATHIC PHYSICIAN:

### THE VACCINATION PAPERS A WASTE OF TIME AND SPACE.

MIAMI, FLORIDA, May 13th, 1898.

EDITOR OF THE HOMŒOPATHIC PHYSICIAN:—You have asked for "a full and free expression of views on the subject" concerning the publication of the papers of Dr. Leverson. I feel that it is not incumbent on me, or in other words, hardly in good taste . . . to in any way suggest or influence you in the direction of your journal. But as a Hahnemannian homœopathist, I do not see that it is doing any good. No physician of our school who is worthy of the rank need be furnished evidence of the nefarious results of vaccination, or its uselessness. I saw that verified in the army during the war, not only in many others, but in my own person. I furnished this for publication some years ago. If not, I told it repeatedly to my classes in the colleges, and have related it in our local societies. Of course I am not acquainted with your subscribers and readers. I do not know their drift as regards vaccination or Homœopathy, but presumably a majority of them are "sound in the faith." If so, they doubtless feel somewhat as I do, that it a waste of time and space to publish those papers. I know how it is with me: I seldom look at them.

S. Mills Fowler, M. D.

[Dr. Fowler is Professor of Practice of Medicine in Dunham Medical College.—Ed.]

